# Reliability of Symbolic Analysis of Heart Rate Variability and Its Changes During Sympathetic Stimulation in Elite Modern Pentathlon Athletes: A Pilot Study

**DOI:** 10.3389/fphys.2022.829887

**Published:** 2022-02-28

**Authors:** Jakub S. Gąsior, Maciej Rosoł, Marcel Młyńczak, Andrew A. Flatt, Bartosz Hoffmann, Rafał Baranowski, Bożena Werner

**Affiliations:** ^1^Department of Pediatric Cardiology and General Pediatrics, Medical University of Warsaw, Warsaw, Poland; ^2^Faculty of Mechatronics, Institute of Metrology and Biomedical Engineering, Warsaw University of Technology, Warsaw, Poland; ^3^Biodynamics and Human Performance Center, Department of Health Sciences and Kinesiology, Georgia Southern University (Armstrong Campus), Savannah, GA, United States; ^4^Physiotherapy Division, Faculty of Medical Sciences, Medical University of Warsaw, Warsaw, Poland; ^5^Department of Heart Rhythm Disorders, National Institute of Cardiology, Warsaw, Poland

**Keywords:** symbolic dynamics, heart rate variability, non-linear, athletes, modern pentathlon athletes

## Abstract

**Background and Purpose:**

Most studies on heart rate variability (HRV) in professional athletes concerned linear, time-, and frequency-domain indices, and there is lack of studies on non-linear parameters in this group. The study aimed to determine the inter-day reliability, and group-related and individual changes of short-term symbolic dynamics (SymDyn) measures during sympathetic nervous system activity (SNSa) stimulation among elite modern pentathletes.

**Methods:**

Short-term electrocardiographic recordings were performed in stable measurement conditions with a 7-day interval between tests. SNSa stimulation *via* isometric handgrip strength test was conducted on the second day of study. The occurrence rate of patterns without variations (0V), with one variation (1V), two like (2LV), and two unlike variations (2UV) obtained using three approaches (the Max–min, the *σ*, and the Equal-probability methods) were analyzed. Relative and absolute reliability were evaluated.

**Results:**

All SymDyn indices obtained using the Max–min method, 0V, and 2UV obtained using the *σ* method, 2UV obtained using the Equal-probability method presented acceptable inter-day reliability (the intraclass correlation coefficient between .91 and .99, Cohen’s *d* between −.08 and .10, the within-subject coefficient of variation between 4% and 22%). 2LV, 2UV, and 0V obtained using the Max–min and *σ* methods significantly decreased and increased, respectively, during SNSa stimulation—such changes were noted for all athletes. There was no significant association between differences in SymDyn parameters and respiratory rate in stable conditions and while comparing stable conditions and SNSa stimulation.

**Conclusion:**

SymDyn indices may be used as reliable non-respiratory-associated parameters in laboratory settings to detect autonomic nervous system (ANS) activity modulations in elite endurance athletes. These findings provide a potential solution for addressing the confounding influence of respiration frequency on HRV-derived inferences of cardiac autonomic function. For this reason, SymDyn may prove to be preferable for field-based monitoring where measurements are unsupervised.

## Introduction

Comprehensive monitoring and identification of the physiological state of athletes by coaches, exercise scientists, and/or sports physicians can be accomplished using sensitive, non-invasive, time-efficient, and cost-effective testing methods and biomarkers. Knowledge of status is important for optimizing training adaptations and improving performance. Status markers may also aid in the diagnosis and risk prediction of medical conditions, such as sport-related concussion in elite athletes (Meeusen et al., 2013; Bourdon et al., 2017; Gabbett et al., 2017; Bishop et al., 2018; Heidari et al., 2018; Kellmann et al., 2018; Schneider et al., 2018). To distinguish between intended (e.g., training-related, lifestyle-related, or injury, e.g., concussion-induced) and unintended (measurement error) changes in selected physiological parameters, it is crucial to use objective and reliable measurements with validated tools (Lachin, 2004; Matheson, 2019).

Heart rate variability (HRV) parameters are becoming increasingly popular as non-invasive, reliable and sensitive biomarkers reflecting changes in autonomic nervous system (ANS) activity in athletes (Baumert et al., 2006; Buchheit, 2014; Flatt and Esco, 2016; Pereira et al., 2016; Esco et al., 2018; Schneider et al., 2018; Vescovi, 2019; Perrone et al., 2021). A vast majority of studies on HRV in professional athletes concerned only linear (time- and frequency-domain) parameters, which may not provide comprehensive description of ANS activity within this populations. Importantly, selected linear HRV parameters are inadequate to assess autonomic control and activity during short-time series commonly observed in exercise physiology or sports medicine (Gronwald et al., 2020; Rogers et al., 2021a,b). The signal stationarity and “controlled” respiratory rate are required for the short-term frequency-domain HRV analysis (Magagnin et al., 2011; Quintana et al., 2016). Therefore, in studies where the immediate effect is measured or specific exercise task influences breathing pattern, these measures are inappropriate. In recent years, measures of the non-linear dynamics of HRV have provided new opportunities to monitor cardiac autonomic regulation (Voss et al., 2009; Sassi et al., 2015; Henriques et al., 2020). It is known that cardiovascular control involves non-linear interactions between physiological systems, with complex dynamics (Schiecke et al., 2018; Saul and Valenza, 2021). Considering that many biomarkers are non-stationary signals (Goldberger, 1991; Faust et al., 2004) and vary in a complex and non-linear way (Voss et al., 2009), some authors highlight that non-linear HRV indices are more suitable than linear for the evaluation of individual changes in test–retest studies (Maestri et al., 2007) and underline the need to perform non-linear analysis to provide holistic information on HRV (Acharya et al., 2006; Huikuri et al., 2009; Voss et al., 2009; Hoshi et al., 2013; Sassi et al., 2015).

Recently (2020), we found that short-term (5-min) non-linear indices of entropy measures (Approximate Entropy and Sample Entropy), Poincaré plot (SD2/SD1), and index of HRV based on fractal correlation properties (short-term scaling exponent of detrended fluctuation analysis) presented large relative or high absolute test–retest reliability among elite endurance athletes (Hoffmann et al., 2020). Apart from these indices, symbolic dynamics have also gained wide acceptance for assessing various complex systems, but their applications in HRV studies are substantially less popular than the aforementioned non-linear parameters (Henriques et al., 2020).

The symbolic analysis method consists mainly of the transformation of a time series (RR intervals) into short patterns (three beats long), their classification, and the evaluation of their rates of occurrence (Porta et al., 2001). Porta et al. (2007a) showed that two non-linear symbolic indexes, that is, patterns with no variation (0V), and patterns with two unlike variations (2UV), represent a valid alternative to linear spectral indexes for assessment of the cardiac autonomic modulation from short-term heart period variability. The enhancement of cardiac sympathetic modulation and reduction of vagal modulation during sympathetic activation was associated with an increase of 0V and a decrease of 2UV (Porta et al., 2007b; Cysarz et al., 2013).

Very recently (2021), Matsumura et al. (2021) showed that sympathetic activity is dominant prior to competition attempts in elite athletes in a real-world sport event and concluded that sympathetic predominance before and/or during professional competition would be advantageous for performance in risky sports, such as snowboard jumping. Recognizing and modifying cardiac sympathetic and parasympathetic activity could be useful for coaches, exercise scientists and/or sports physicians in helping athletes to improve their physical performance and achieve better sport results.

Summarizing, little is known about the reliability of symbolic analysis when applied to short-term measurements in athletic populations (Gronwald and Hoos, 2020; Uhlig et al., 2020). HRV analysis has been mostly performed on the basis of group changes in endurance athletes (Baumert et al., 2006; Schmitt et al., 2018; Deus et al., 2019), which is ineffective in detecting individual athletes’ responses (Muñoz-López and Naranjo-Orellana, 2020). Greater attention to individual analysis seems to be necessary when assessing physiological responses to a specific training or interventions in athletes (Gąsior et al., 2020; Hoffmann et al., 2020). Cardiac autonomic modulation is linked to spontaneous respiration (Eckberg and Eckberg, 1982; Eckberg et al., 1985; Eckberg, 2003; Narkiewicz et al., 2006; Bari et al., 2016), hence, HRV (mostly frequency-domain) parameters are affected by respiratory depth and frequency (Hirsch and Bishop, 1981; Brown et al., 1993; De Souza et al., 2018). Sports field practitioners should interpret changes in HRV parameters with concomitant respiratory rate alterations. Therefore, the presented study has the following aims: (i) to assess the inter-day reliability of symbolic dynamics (SymDyn) indices obtained using three different transformations; (ii) to determine group and individual changes during SNSa stimulation in short-term SymDyn measures; and (iii) to quantify associations between differences in respiratory rate and SymDyn measures among elite modern pentathletes.

## Materials and Methods

**Figure 1:**
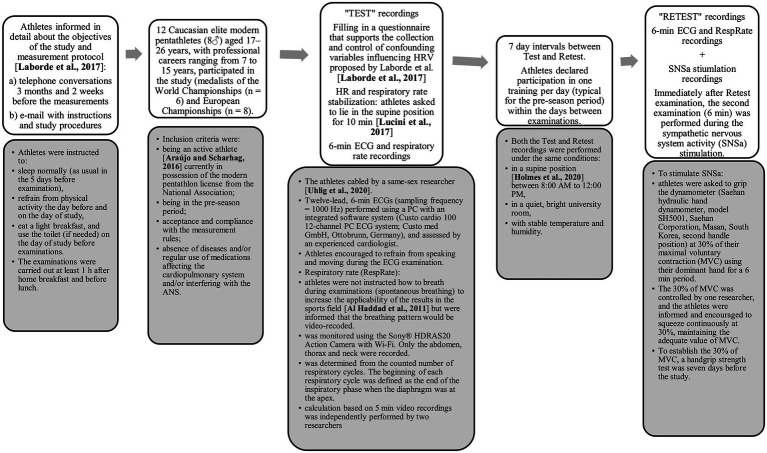
Study protocol.

Study protocol with details is presented in Figure 1. Details of the study population (participants), study protocol, ECG acquisition, respiratory rate recordings, and SNSa stimulation have been presented elsewhere (Gąsior et al., 2020; Hoffmann et al., 2020). The study was approved by the University Ethical Committee (SKE 01-01/2017, 7 March 2017, Warsaw, Poland) and followed the rules and principles of the Helsinki Declaration. All athletes were informed of the aims and risks involved with the protocol and subsequently provided written informed consent prior to data collection.

The ECGs were visually inspected for potential non-sinus or aberrant beats, and such erroneous beats were corrected from the cardiac interval series (RR series) before HRV analysis. The erroneous beats were manually corrected, that is, one R-R interval before, and one after each non-sinus beat were eliminated and replaced by R-R intervals computed by interpolation of degree zero based on the surrounding normal beats (Peltola, 2012).

Screened and corrected 5-min RR series were imported for analysis into PyBiOS software from ASCII text files (Silva et al., 2020). Imported RR series were not corrected for artifacts, segmented, filtered, or detrended using software tools.

### Analysis of Symbolic Dynamics

In general, symbolic analysis consist of three steps: (1) conversion of the original series into a series of symbols; (2) definition of words (sequences of symbols) of interest; and (3) quantification of the different words, quantities that may be related to the phenomena of interest. The second and third steps in three transformation methods (Max–min method, σ method, and Equal-probability method) are similar. They differ in the way the signal of symbols is generated (Cysarz et al., 2018). Details concerning time series transformation into the symbol sequences using the appropriate alphabet for each method were precisely described by Wessel et al. (2007), Porta et al., (2007b) and Cysarz et al., (2013, 2018).

In this study, SymDyn indexes were obtained using three transformation methods: Max–min method, σ method, and Equal-probability method (Cysarz et al., 2013, 2018).

In Max–min method, the series of RR intervals were converted into a series of symbols through a uniform quantization of six levels (quantization level: 6). This means that six equal ranges were defined from the minimum to the maximum value within the series [*l* = (max(*x*_i_)—min(*x*_i_)]/ξ, quantization level *ξ* = 6), and each value in the original series was converted into a symbol (0–5) according to the range it belongs to Porta et al. (2001, 2007b).

In the *σ* method, three levels were defined using the following quantization lines: the signal average (*μ*), the signal average shifted up by a factor a, that is, (1 + *a*) *μ*, and the signal average shifted down by a factor a, that is, (1 − *a*) *μ*. The parameter *a* (sigma rate) was set to .05 (Kurths et al., 1995).

The Equal-probability method divides the full range of the signal into quantization levels ensuring that all levels will contain the same number of points. Thus, if the signal has length *L*, each level will have *L*/quantization level samples. If *L* is not a multiple of quantization level, the number of points can vary by one within the levels (Cysarz et al., 2018). The transformation was used with two different quantization levels: 4 (*q* = 4) and 6 (*q* = 6) enabling a direct comparison with the σ method and Max–min method, respectively.

For all the three methods, all sequences of three consecutive symbols (words) are classified into one of four families: 0V (zero variation), if the three symbols are equal. Examples of words from this family are {1,1,1} and {5,5,5}; 1V (one variation), which represent sequences with only one variation. The words {1,1,2} and {3,3,0} are examples from this family; 2LV (two like variation), representing sequences with two variations in the same direction, that is, the symbols are all different and form an increasing or decreasing ramp. Examples from this family are {0,3,5} and {2,1,0}; 2UV (two unlike variation), where symbols vary two times, in opposite directions, composing a peak or a valley. The words {1,2,0} and {3,0,3} are examples from this family. Finally, the percentages of words classified into each family are taken for analysis of the series dynamics (Porta et al., 2001, 2007a,b; Guzzetti et al., 2005; Cysarz et al., 2013, 2015, 2018).

Evidently, words from 0V family represent the slowest oscillations in the series, whereas words from 2UV represent the fastest. The 1V and 2LV families are intermediate levels. In case of HRV series, higher 0V% and 2UV% were associated with the higher the sympathetic and vagal modulation to the heart, respectively (Porta et al., 2001, 2007a,b, 2015; Guzzetti et al., 2005; Baumert et al., 2009; Tobaldini et al., 2009; Cysarz et al., 2013; Silva et al., 2017).

### Statistical Analysis

For reliability assessment, the intraclass correlation coefficient (ICC), the within-subject coefficient of variation (WSCV) and Cohen’s *d* were calculated. ICC (Shrout and Fleiss, 1979) and WSCV (Bland and Altman, 1996; Atkinson and Nevill, 1998; Hopkins, 2000a,b) were used to analyze the relative reliability and absolute reliability respectively, while Cohen’s *d* was used to assess the effect size of the mean differences between repeated measurements. The interpretation of ICC values was determined *a priori* as follows: from 0 to .30—small, from .31 to .49—moderate, from .50 to .69—large, from .70 to .89 very large and from .90 to 1.00 the value was considered nearly perfect (Hopkins et al., 2009). Smaller WSCV means better reproducibility (Shoukri et al., 2008). The interpretation of Cohen’s *d* (Cohen, 1988) was also determined *a priori*, considering the value less than .20 as trivial, from .21 to .60 as small, from .61 to 1.20 as moderate, from 1.21 to 2.00 as large and greater than 2.01 as very large (Hopkins et al., 2009). Overall, if ICC was greater than .90 and Cohen’s *d* value trivial, this was interpreted as acceptable reliability. For visualization of the data, Bland–Altman plots were created with marked 95% limits of agreement (LoA) and maximum allowed difference (Abu-Arafeh et al., 2016), which was equal to the smallest worthwhile change (SWC) calculated as a .2 times SD of the Test measurements values (Buchheit, 2018).

In order to compare the results obtained from test and retest, Wilcoxon signed-rank tests were used (due to the small sample size). To assess the correlation between repeated measurements, Spearman’s rank correlation coefficient, Lin’s concordance coefficient, and bias correction factor (from Lin’s analysis), were calculated. Also, the dependency between the obtained results was evaluated by intercept from linear regression models. The ratio of the intercept from the model, divided by the mean value of the parameter from both measurements, enables the relative comparison of the results for individual parameters. The obtained value is associated with the bias for the consecutive measurements.

To compare values from Retest and SNSa stimulation, and hence the impact of the intervention on the parameter values, the mean and SD of the difference between measurements were calculated. Then, those values were divided by the mean value of the given parameter in both groups and presented as a percentage rate of change to enable the comparison of the influence of the intervention on the results. To compare the results from Retest and SNSa stimulation, Wilcoxon signed-rank tests were performed. Bland–Altman plots were also created for each comparison.

The changes in HRV parameter values between two measurements (test–retest and retest-SNSa) were studied in association with the changes in respiratory rates (identically like presented elsewhere Hoffmann et al., 2020; Gąsior et al., 2020). Correlation plots, along with linear regression model fitting were created for consecutive measurements with the results of Pearson correlation coefficients (*r*) for each of those associations. In this and other analyses, the change or difference of the parameters was studied by subtracting the values for Test from Retest, and values for Retest from SNSa.

Analysis based on group changes is ineffective in detecting individual athletes’ responses (Muñoz-López and Naranjo-Orellana, 2020). It was suggested that HRV be assessed in athletes on an individual basis (Plews et al., 2013). Therefore, individual athletes’ responses have been graphically presented in this study.

Statistical analysis and figures were, respectively, performed and prepared using Python 3.7.10 (including primarily modules: NumPy version 1.18.1, Pandas 1.0.1, SciPy 1.4.1 and scikit-learn version 0.22.1) and R 4.1.0 (including primarily packages: irr version 0.84.1 and effsize version 0.8.1). The significance level is .05.

## Results

Results of four participants out of 12 were excluded due to diagnosis of prolonged QTc interval >450 ms (*n* = 2) or left bundle branch block (*n* = 2)—athletes were referred for further medical investigation. Results of eight male active elite modern Caucasian pentathletes living in Warsaw (Poland), medallists of the World Championships (*n* = 3), European Championships (*n* = 5), and National Championships (*n* = 8) were included in the statistical analysis. The mean (±SD) age, weight, height, body mass index (BMI), and duration of professional athletic career were: 21.7 years (±3.1), 75.9 kg (±9.5), 182.6 cm (±6.1), 22.7 kg/m^2^ (±2.3), and 10.8 years (±2.9). Athletes declared participating in 19 training sessions (±2) per week during the normal in-season time.

### Reliability Analysis and Differences Between Test and Retest

Results for reliability analysis are presented in Table 1. For all SymDyn indices obtained using the Max–min method, 0V and 2UV obtained using the σ method, and 2UV obtained using the Equal-probability methods, ICC and Cohen’s *d* were nearly perfect and trivial, respectively. WSCV was between 2% and 12% for all SymDyn indices obtained using the Max–min method, between 8% and 25% using the σ-method, between 7% and 28% using the Equal-probability methods.

**Table 1:** Results of reliability analysis.

Parameter		ICC (95% CI)	WSCV (%) (95% CI)	Cohen’s *d* (95% CI)
Max–min method	0V	.95 (.80–.99)	11.8 (7.9–15.7)	.03 (−1.04–1.11)
	1V	.91 (.64–.98)	2.3 (2.1–2.5)	−.08 (−1.15–.99)
	2LV	.95 (.78–.99)	11.4 (7.7–15.1)	.10 (−.98–1.17)
	2UV	.99 (.96–1.00)	4.3 (2.7–2.9)	.02 (−1.06–1.09)
*σ* method	0V	.91 (.64–.98)	21.9 (11.1–32.6)	.06 (−1.01–1.14)
	1V	.47 (−.24–.86)	7.6 (6.3–8.9)	−.33 (−1.41–.75)
	2LV	.59 (−.08–.90)	24.7 (10.3–39.0)	.42 (−.68–1.50)
	2UV	.94 (.77–.99)	17.6 (11.5–23.7)	.10 (−.97–1.17)
Equal-probability method (*q* = 6)	0V	.78 (.29–.95)	28.0 (13.3–42.8)	.05 (−1.03–1.12)
	1V	.18 (−.52–.75)	16.9 (12.3–21.4)	−.83 (−1.95–.29)
	2LV	.51 (−.20–.87)	18.9 (7.3–30.5)	.19 (−.89–1.26)
	2UV	.95 (.80–.99)	16.5 (7.7–25.4)	.08 (−.99–1.15)
Equal-probability method (*q* = 4)	0V	.72 (.15–.94)	27.2 (16.7–37.6)	.06 (−1.01–1.13)
	1V	.71 (.13–.93)	7.4 (5.3–9.6)	−.07 (−1.14–1.00)
	2LV	.56 (−.13–.89)	22.7 (3.5–41.8)	.14 (−.93–1.22)
	2UV	.96 (.84–.99)	15.0 (6.4–23.5)	.07 (−1.00–1.14)

q, quantization level; 0V, patterns with no variation; 1V, patterns with one variation; 2LV, patterns with two like variations; 2UV, patterns with two unlike variations; ICC, intraclass correlation coefficient; WSCV, within-subject coefficient of variation; and CI, confidence interval.

**Figure 2:**
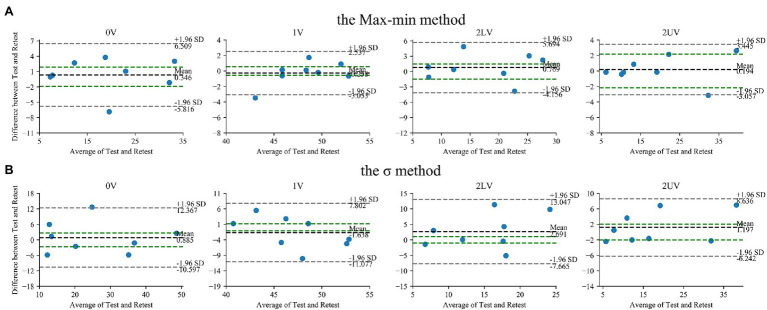
Bland–Altman plots for SymDyn indices: 0V, 1V, 2LV, and 2UV (Test and Retest): **(A)** the Max–min method, **(B)** the *σ*-method. The black dashed line indicates the mean difference, the grey dashed lines are the limits of agreement (±1.96 SD), and the green lines are the maximum allowed difference.

**Figure 3:**
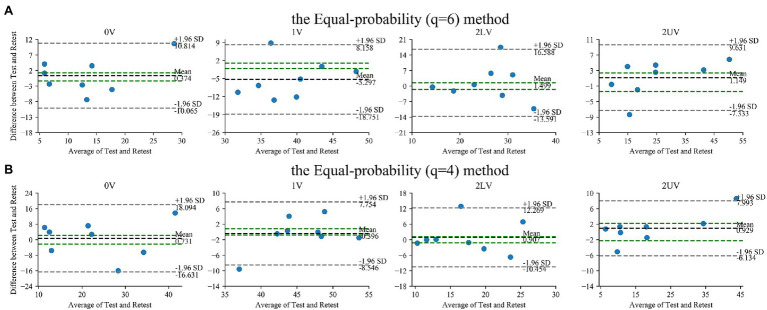
Bland–Altman plots for SymDyn indices: 0V, 1V, 2LV, and 2UV (Test and Retest): the Equal-probability method **(A)** *q* = 6, **(B)** *q* = 4. The black dashed line indicates the mean difference, the grey dashed lines are the limits of agreement (±1.96 SD), and the green lines are the maximum allowed difference.

The Bland–Altman plots for SymDyn indices obtained using the Max–min method, the *σ* method, and the Equal-probability method *q* = 6, *q* = 4 presented in Figures 2, 3, respectively. The smallest mean difference between Test and Retest was observed for SymDyn indices obtained using the Max–min method. The maximum allowed differences (i.e., smallest worthwhile changes) for 0V, 1V, 2LV, and 2UV were: 1.9, .5, 1.5, 2.2 (the Max–min method); 2.6, 1.1, 1.0, 2.0 (the *σ* method); 1.3, 1.0, 1.5, 2.4 (the Equal-probability method *q* = 6); 2.2, .8, 1.2, 2.2 (the Equal-probability method *q* = 4), respectively. For all analyzed parameters, regardless of the method used, LoA exceeded the defined maximum allowed difference.

There were no significant differences in all SymDyn indices obtained using all methods between Test and Retest. There was a significant correlation between values from Test and Retest for all indices obtained using the Max–min method, for 0V and 2UV obtained using the σ method and the Equal-probability method (*q* = 6), for 1V and 2UV obtained using the Equal-probability method (*q* = 4). Lin’s concordance coefficient was the highest for 2UV regardless of the method used. For the rest of SymDyn indices obtained using the Max–min method, Lin’s concordance coefficient was between .89 and .94. The smallest value of the intercept of the linear model scaled by the mean value of the parameter was observed for 2UV obtained using the Max–min method, while the highest for 1V obtained using the σ method (Table 2).

**Table 2:** Results of the comparison between Test and Retest in analyzed symbolic dynamics indices.

Parameter		Test (mean ± SD)	Retest (mean ± SD)	*p*	Spearman correlation coefficient (*p*)	Lin’s concordance coefficient	Bias correction factor from Lin’s analysis	Intercept of the linear model divided by the mean value
Max–min method	0V	19.1 ± 9.5	19.4 ± 9.5	.48	.90 (<.01)	.94	1.00	.06
	1V	48.4 ± 2.7	48.1 ± 3.5	.78	.90 (<.01)	.89	.96	−.21
	2LV	16.9 ± 7.4	17.6 ± 7.5	.41	.93 (<.001)	.94	.99	.08
	2UV	19.1 ± 10.8	19.3 ± 11.1	1.00	.99 (<.001)	.99	1.00	<.001
*σ* method	0V	25.1 ± 13.0	25.9 ± 12.8	.78	.86 (<.01)	.90	1.00	.15
	1V	48.1 ± 5.6	46.4 ± 3.4	.48	.60 (.12)	.43	.83	.67
	2LV	13.7 ± 5.0	16.4 ± 6.9	.33	.55 (.16)	.56	.87	.28
	2UV	17.2 ± 10.2	18.4 ± 11.7	.58	.95 (<.001)	.94	.98	−.02
Equal-probability method (*q* = 6)	0V	12.9 ± 6.5	13.3 ± 8.6	1.00	.74 (<.05)	.76	.96	−.01
	1V	41.6 ± 5.2	36.3 ± 6.7	.07	.45 (.26)	.24	.69	.45
	2LV	25.0 ± 7.6	26.5 ± 7.3	.67	.38 (.35)	.46	.98	.59
	2UV	24.3 ± 12.1	25.5 ± 14.5	.33	.90 (<.01)	.94	.98	−.11
Equal-probability method (*q* = 4)	0V	22.7 ± 11.1	23.4 ± 11.3	.78	.69 (.06)	.69	1.00	.32
	1V	45.9 ± 4.1	45.5 ± 6.1	.67	.83 (<.01)	.68	.93	−.08
	2LV	16.7 ± 5.8	17.7 ± 5.9	.89	.45 (.26)	.51	.99	.52
	2UV	18.5 ± 11.1	19.4 ± 13.9	.48	.88 (<.01)	.96	.97	−.17

q, quantization level; 0V, patterns with no variation; 1V, patterns with one variation; 2LV, patterns with two like variations; 2UV, patterns with two unlike variations; and SD, standard deviation.

### SNSa Stimulation (Impact of Intervention)

**Figure 4:**
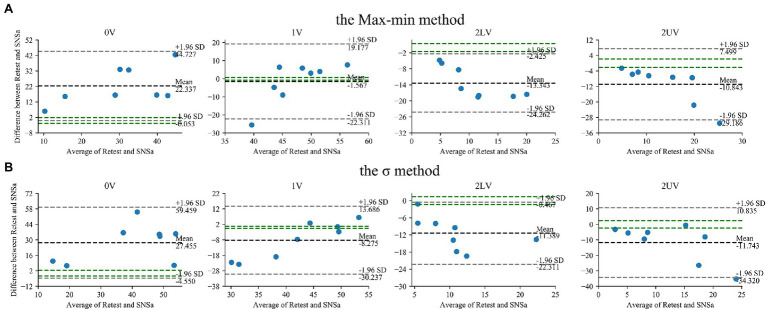
Bland–Altman plots for SymDyn indices 0V, 1V, 2LV, and 2UV (Retest and sympathetic nervous system activity—SNSa stimulation): **(A)** the Max–min method, **(B)** the *σ*-method. The black dashed line indicates the mean difference, the grey dashed lines are the limits of agreement (±1.96 SD), and the green lines are the maximum allowed difference.

**Figure 5:**
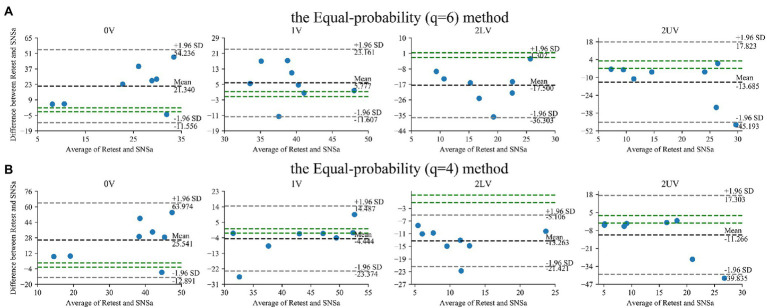
Bland–Altman plots for SymDyn indices 0V, 1V, 2LV, and 2UV (Retest and SNSa stimulation): the Equal-probability method **(A)** *q* = 6, **(B)** *q* = 4. The black dashed line indicates the mean difference, the grey dashed lines are the limits of agreement (±1.96 SD), and the green lines are the maximum allowed difference.

Statistically significant increases in 0V and decreases in both 2LV and 2UV with no changes in 1V were observed during SNSa stimulation, independently on the method used to obtain indices (Table 3). The largest percentage change was observed for 2LV. The Bland–Altman plots for SymDyn indices obtained using the Max–min method, the σ method and the Equal-probability method *q* = 6, *q* = 4 are presented in Figures 4, 5, respectively. The smallest mean difference between Retest and SNSa was observed for 1V obtained using all methods.

**Table 3:** Results of the comparison between Retest and SNSa stimulation in analyzed symbolic dynamics indices.

Parameter		Retest (mean ± SD)	SNSa stimulation (mean ± SD)	*p*	Mean of the difference divided by the mean value (%)	SD of the difference divided by the mean value (%)
Max–min method	0V	19.4 ± 9.5	41.8 ± 15.6	.01	73.0	37.3
	1V	48.1 ± 3.5	46.6 ± 9.6	1.00	−3.3	22.3
	2LV	17.6 ± 7.5	4.3 ± 3.4	.01	−121.8	50.8
	2UV	19.3 ± 11.1	8.4 ± 3.8	.01	−78.3	67.6
*σ* method	0V	25.9 ± 12.8	53.4 ± 19.3	.01	69.2	41.2
	1V	46.4 ± 3.4	38.1 ± 13.4	.16	−19.6	26.5
	2LV	16.4 ± 6.9	5.0 ± 4.2	.01	−106.3	52.0
	2UV	18.4 ± 11.7	6.6 ± 4.9	.01	−94.0	92.2
Equal-probability method (*q* = 6)	0V	13.3 ± 8.6	34.6 ± 15.1	.02	89.1	70.1
	1V	36.3 ± 6.7	42.1 ± 5.3	.09	14.7	22.6
	2LV	26.5 ± 7.3	9.0 ± 7.2	.01	−98.6	54.0
	2UV	25.5 ± 14.5	11.8 ± 7.5	.02	−73.5	86.4
Equal-probability method (*q* = 4)	0V	23.4 ± 11.3	49.0 ± 18.3	.02	70.6	54.2
	1V	45.5 ± 6.1	41.1 ± 11.7	.12	−10.3	22.3
	2LV	17.7 ± 5.9	4.4 ± 5.6	.01	−120.3	37.8
	2UV	19.4 ± 13.9	8.2 ± 5.0	.01	−81.7	105.7

q, quantization level; 0V, patterns with no variation; 1V, patterns with one variation; 2LV, patterns with two like variations; 2UV, patterns with two unlike variations; SD, standard deviation; and SNSa, sympathetic nervous system activity.

### Correlation Between Changes in Non-linear HRV Parameters and Changes in RespRate

**Figure 6:**
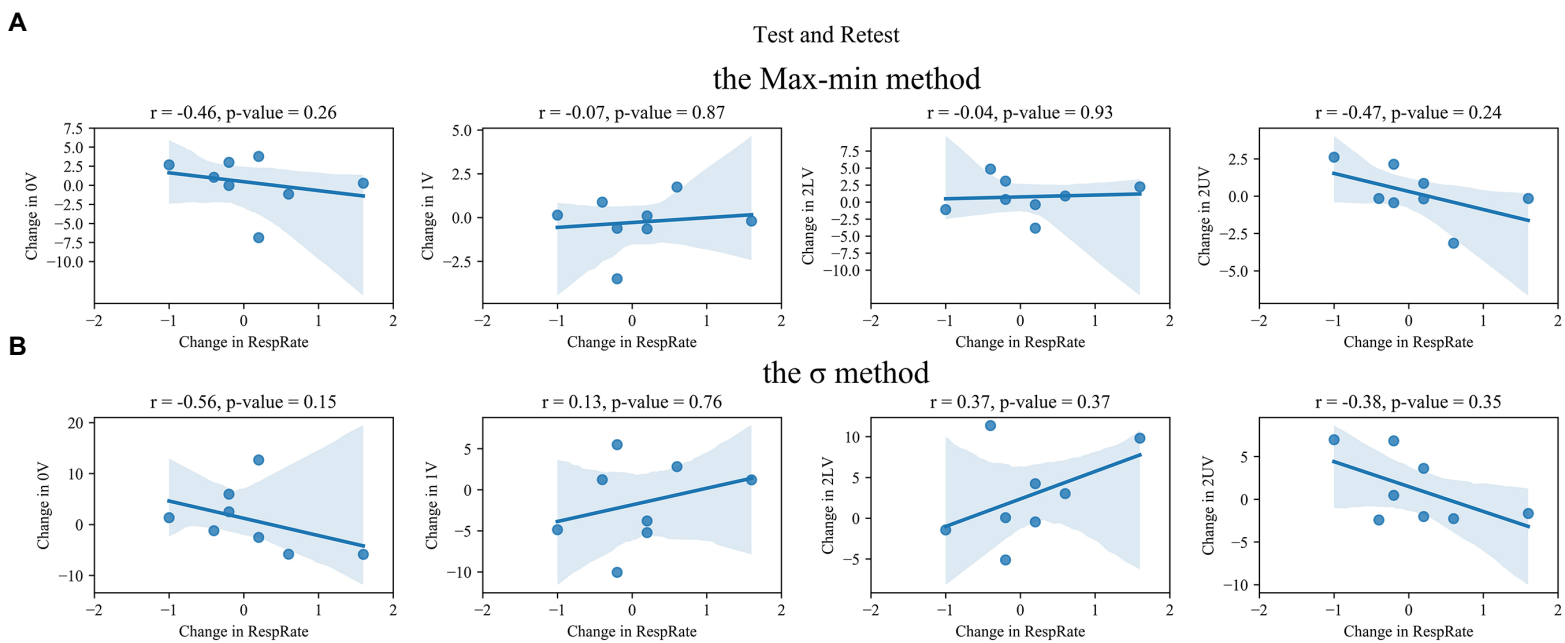
Correlation between retest–test differences in SymDyn indices and differences in respiratory rate (RespRate): **(A)** the Max–min method, **(B)** the *σ*-method.

**Figure 7:**
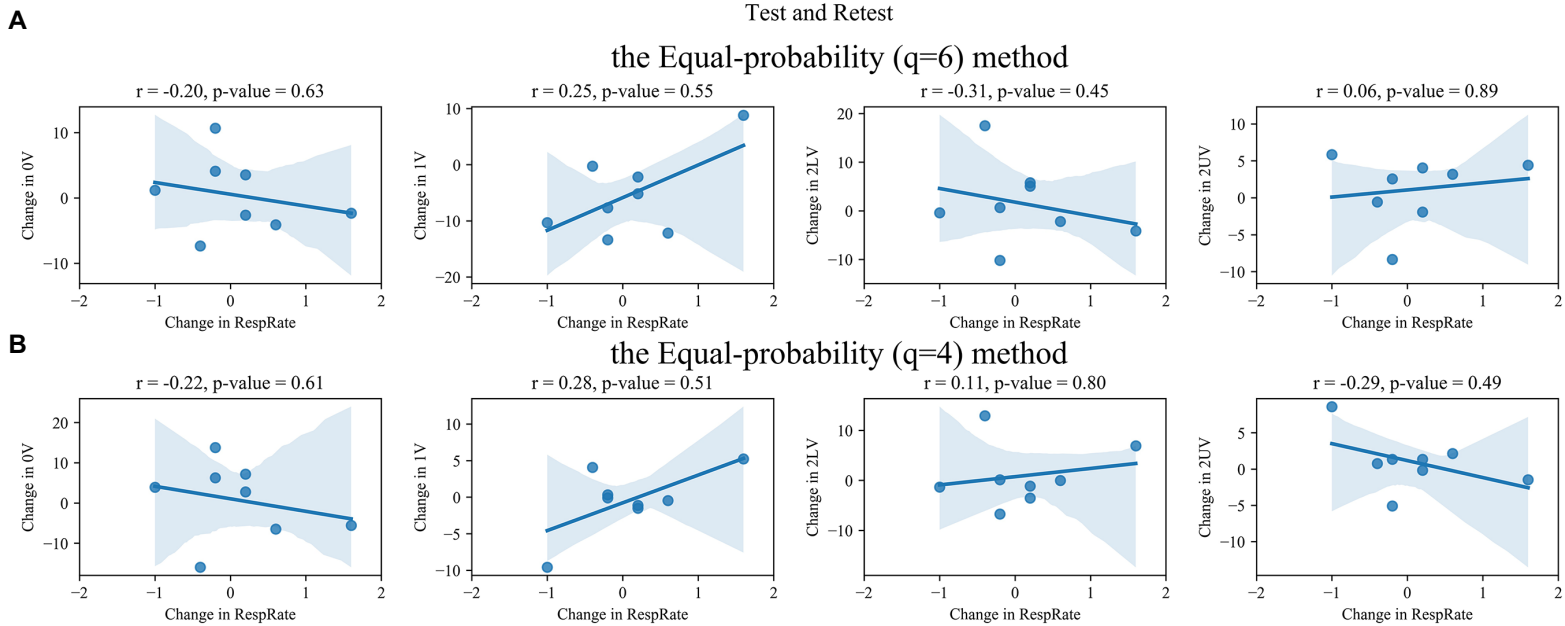
Correlation between retest–test differences in SymDyn indices and differences in respiratory rate (RespRate): the Equal-probability method **(A)** *q* = 6, **(B)** *q* = 4.

**Figure 8:**
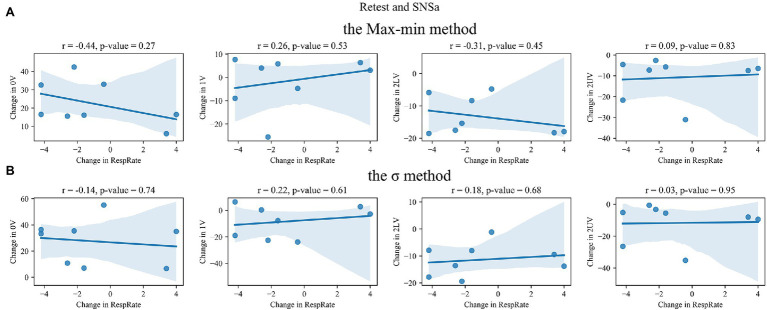
Correlation between SNSa stimulation–retest differences in SymDyn indices and differences in respiratory rate (RespRate): **(A)** the Max–min method, **(B)** the *σ*-method.

**Figure 9:**
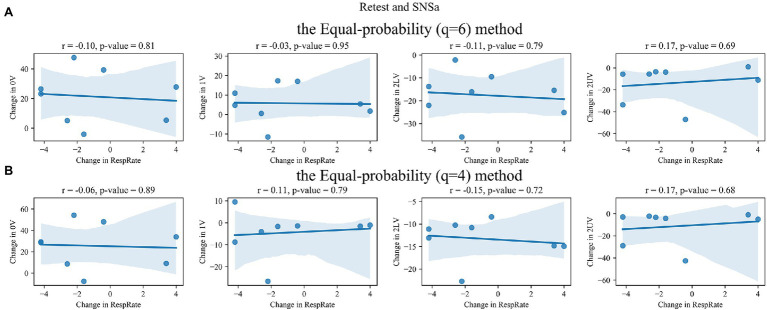
Correlation between SNSa stimulation–retest differences in SymDyn indices and differences in respiratory rate (RespRate): the Equal-probability method **(A)** *q* = 6, **(B)** *q* = 4.

The correlations for retest–test and SNSa stimulation–retest differences between SymDyn indices and RespRate are shown in Figures 6–9, respectively. The correlation coefficients were not significant for all indices for all methods.

### Individual Changes

**Figure 10:**
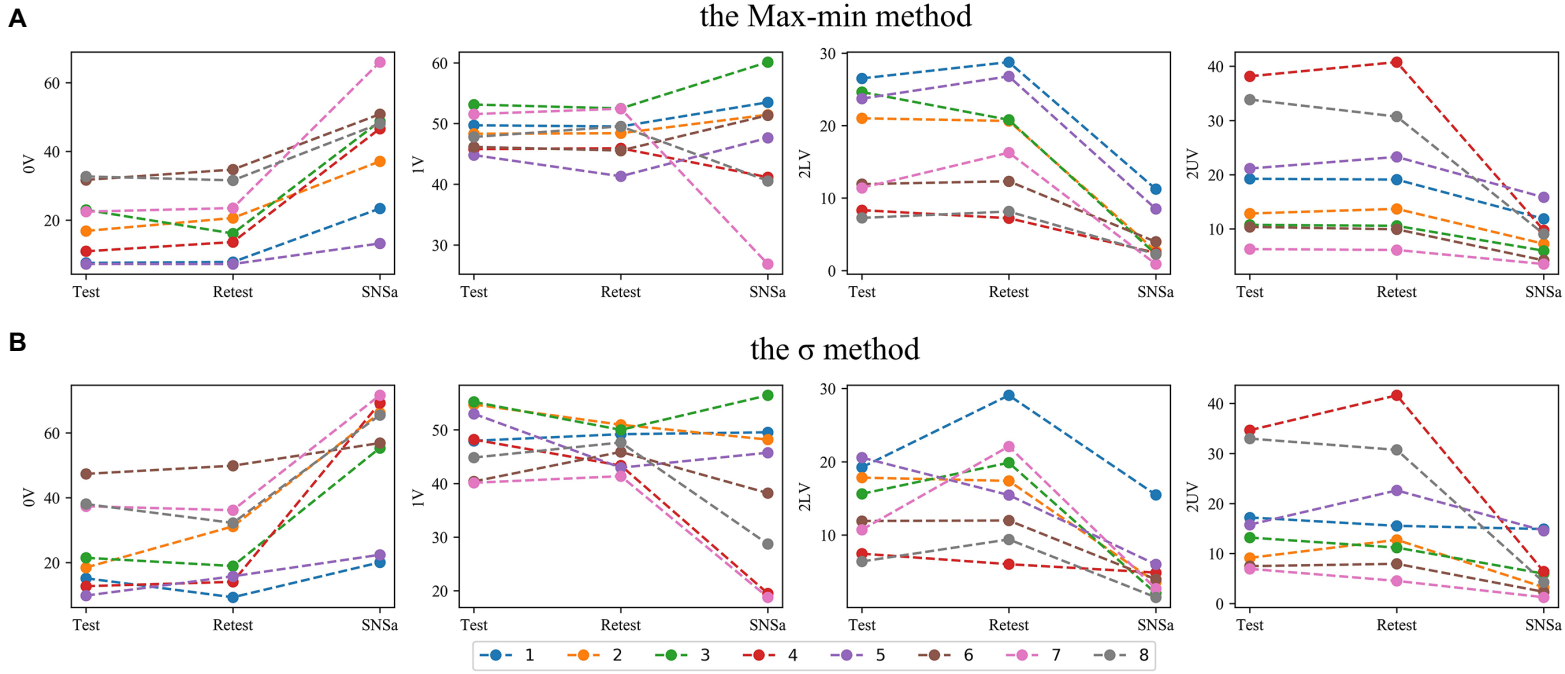
Individual changes in SymDyn indices between Test, Retest, and SNSa stimulation: **(A)** the Max–min method, **(B)** *σ*-method.

**Figure 11:**
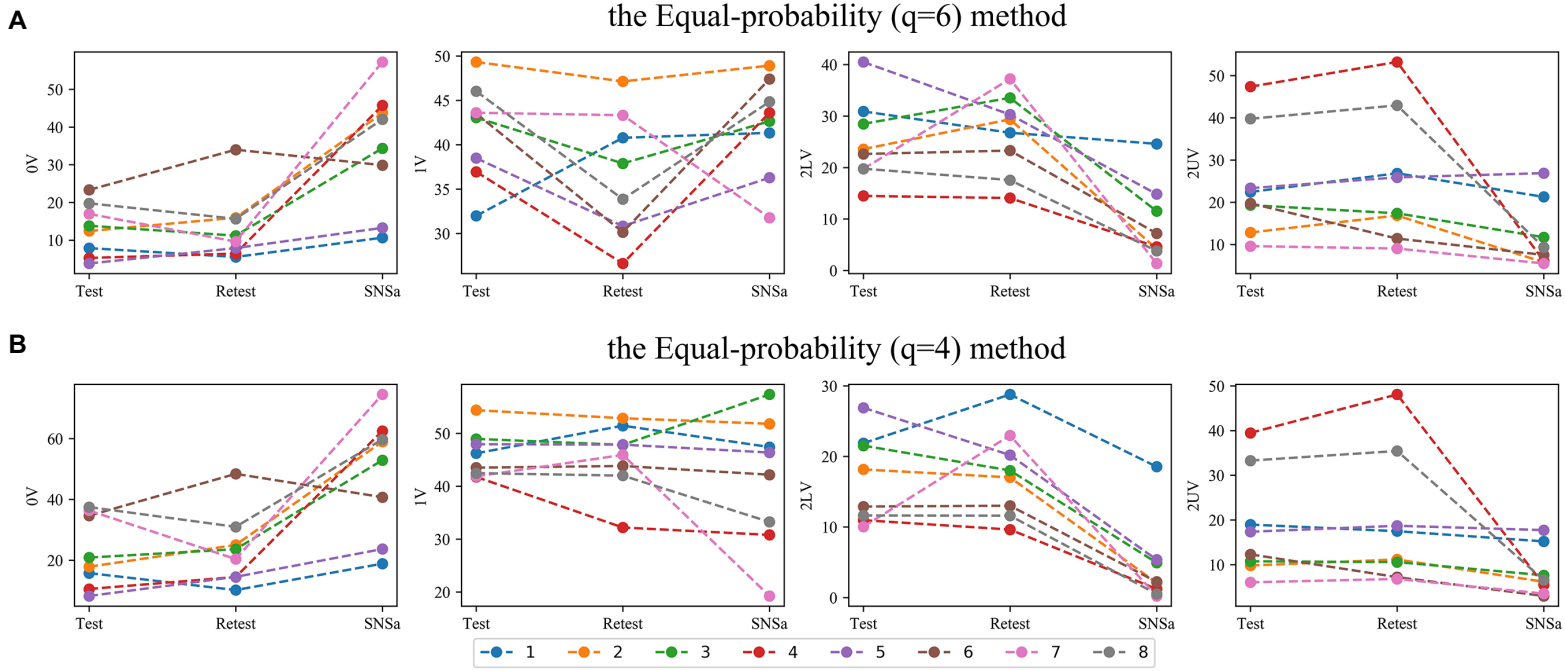
Individual changes in SymDyn indices between Test, Retest, and SNSa stimulation: the Equal-probability method **(A)** *q* = 6, **(B)** *q* = 4.

By analyzing individual HRV changes, professionals can identify athletes who show different responsiveness to the selected stressors (Mann et al., 2014; Vescovi, 2019; Muñoz-López and Naranjo-Orellana, 2020). SymDyn indices values for individuals during subsequent measurements are shown in Figure 10 for the Max–min method and *σ* method and in Figure 11 for the Equal-probability methods. SymDyn indices obtained using the Max–min method and the σ method presented a similar pattern of changes, that is, increase in 0V and decrease in 2LV, 2UV during SNSa stimulation were observed for all athletes (Figure 10). For indices obtained using the Equal-probability methods, there was one athlete (#6) who presented a lower value of 0V during SNSa stimulation than in Retest and one athlete (#5) who had a higher value of 2UV during SNSa than in Retest (but only for the Equal-probability method *q* = 6; Figure 11). In all other athletes, the same pattern was observed as described for the Max–min and the σ methods. The highest variations in individual changes were observed for 1V.

## Discussion

All symbolic dynamics measures obtained using the Max–min method, 0V and 2UV obtained using the σ method, and 2UV obtained using the Equal-probability methods, calculated based on short-term (5-min) RR series derived from ECG recordings performed in stable measurement conditions with 1-week time interval between examinations presented acceptable inter-day reliability in elite modern pentathletes. We found that the rate of patterns with two variations (2LV and 2UV) and the frequency of patterns with no variations (0V) obtained using the Max–min and *σ* methods significantly decreased and increased, respectively, during SNSa stimulation. Such changes were noted for all athletes. The rate of patterns with one variation (1V) remained unchanged with different responses among athletes. There was no significant association between differences in SymDyn parameters and respiratory rate in stable conditions and while comparing stable conditions and SNSa stimulation.

To summarize findings from our research project, short-term (5-min) time-domain log transformed RMSSD (ICC = .98, Cohen’s *d* = .11, WSCV = 2%), frequency-domain log transformed HF (ICC = .86, Cohen’s *d* = .17, WSCV = 6%), descriptors of the Poincaré plot—SD2/SD1 (ICC = .87, Cohen’s *d* = −.07, WSCV = 9%; Hoffmann et al., 2020), 0V obtained using the Max–min and the *σ* method (ICC between .91 and .95, Cohen’s *d* between .03 and .06, WSCV between 12% and 22%) and 2UV obtained using all methods (ICC between .94 and .99, Cohen’s *d* between .02 and .10, WSCV between 4% and 18%) may be used by sport practitioners and researchers as reliable parameters in elite modern pentathletes to detect changes during baseline examinations in laboratory settings. Good absolute and substantial relative reliability for SymDyn variable (2UV) obtained from laboratory recordings was confirmed for healthy volunteers by Maestri et al. (2007).

Sport professionals should keep in mind that some athletes may present values during re-examination in the same stable conditions that are higher or lower than maximum acceptable difference (smallest worthwhile change). Recent reviews have underlined that important data from the Bland–Altman method are often omitted (Abu-Arafeh et al., 2016; Gerke, 2020). The definition of the *a priori* acceptable LoA, to define the minimal agreement between measurement was set as the first key item from comprehensive list of items published in Abu-Arafeh et al. (2016). In our study, all analyzed SymDyn indices showed a LoA that exceeded the defined *a priori* maximum acceptable difference (SWC).

Significant correlations between test–retest differences in respiratory rate and HF in elite endurance athletes were shown (Hoffmann et al., 2020). In presented results, there was no significant association between test–retest differences in respiratory rate and SymDyn indices. Non-linear HRV measures do not require a stationary signal (Michael et al., 2017). Lack of significant dependency on respiratory rate may be considered as another advantage of SymDyn indices. However, alterations in SymDyn parameters may be associated with respiratory depth. In some applications depth of breathing appeared more important for the relation with HRV parameters that its rate (Młyńczak and Krysztofiak, 2019). There is no state-of-the-art solution on how to record respiratory mechanics along with cardiac activity in HRV studies (Quintana and Heathers, 2014; De Souza et al., 2018). One possible solution would be to use Pneumonitors—portable devices measuring both depth and rate of breathing using impedance pneumography, and single-lead ECG (designed specifically for environmental physiology and sports medicine analysis; Młyńczak et al., 2014, 2017).

Porta et al. (2007b) and Cysarz et al. (2013) performed a head-up tilt test to assess cardiac autonomic regulation by symbolic indexes obtained from short-term recordings (250 cardiac beats; range 220–260) in healthy subjects. It was shown that SymDyn indexes are linearly associated with tilt angles during the test (the correlation was observed in more participants for 0V and 2UV than for any spectral parameters) and represent different aspects of the autonomic response to head-up tilt test. Authors suggested that symbolic indexes might be considered as an alternative to frequency-domain HRV parameters in scenarios where short-term recordings are analyzed (Porta et al., 2007b). One of the key differences between these two approaches is that spectral analysis is bounded to long sinusoid components, with different frequencies (in Hz), whereas the SymDyn decomposes the series into small sequences (three values), and the oscillatory patterns are not restricted to any frequency in Hz.

Very recently (2021) Storniolo et al. (2021) analyzed results of symbolic markers pre, post, and during a plateau phase—very short epoch (22 ± 8 cardiac beats, corresponding to 9 ± 3 s.) following maximal sprint exercise—in physically active subjects. Authors observed increased 2UV% and unvaried 0V% during plateau compared to pre sequences, suggesting a remarkable vagal modulation and persistency of the sympathetic control, respectively; 0V% significantly decreased and 2UV% was significantly higher in post compared to pre sequences, suggesting a less active sympathetic and more active vagal control in post than pre sequences, respectively (Storniolo et al., 2021). Authors underlined that such conclusions cannot be achieved from the LF/HF ratio parameter. Moreover, they suggested that future studies should evaluate the eventual association of the symbolic measures with the athlete’s performance (Storniolo et al., 2021).

In our study, to stimulate and assess cardiovascular autonomic function and responsiveness in elite endurance athletes, the isometric handgrip strength test was used (Khurana and Setty, 1996). The effect and procedure of squeezing the dynamometer’s handle may be, to some extent, considered as pre-competition stress and comparable in sports like modern pentathlon to shooting performance. During SNSa stimulation, not unexpectedly, 0V and 2UV were, respectively, higher and lower compared to baseline examination, suggesting more active sympathetic and less active vagal control. Importantly, such changes of these SymDyn indexes (calculated using the Max–min and *σ* methods) were observed for all athletes. Successful detecting and recognizing individual athletes’ cardiac autonomic activity could be helpful in achieving better sport results as it was shown that, for example, sympathetic predominance prior and/or during competition could be advantageous for performance in extreme sports professional athletes (Matsumura et al., 2021). Analysis of inter-individual responses of modifiable biomarkers to a specified stimulus may help in the identification of athletes that will benefit from practical techniques aimed at avoiding pre-performance stress and improve performance in sports.

To monitor athletes in the field using HRV indices Buchheit discouraged practitioners using spectral indices and recommended more appropriate linear time-domain parameters (RMSSD or SD1 reflected parasympathetic modulation) due to their calculation simplicity (using, e.g., an Excel spreadsheet) based on short-term or ultra-short-term recordings and very low sensitivity to breathing pattern (Buchheit, 2014). SymDyn indices may be a good alternative for frequency-domain parameters: 0V and 2UV have been demonstrated to be correlated with the sympathetic and parasympathetic autonomic modulation to the heart (Porta et al., 2001, 2007a,b; Guzzetti et al., 2005; Baumert et al., 2009; Tobaldini et al., 2009; Cysarz et al., 2013, 2018; Silva et al., 2017), can be easily calculated based on very short-term recordings (Storniolo et al., 2021) using recently presented, available free of charge, software tool—PyBioS (Silva et al., 2020).

Small sample size of only male participants, cross-sectional study design, data obtained during supine ECG recordings performed in controlled laboratory settings and lack of additional collection of RR intervals using HR monitor to provide more practical aspect of sports field should be recognized as limitations of this pilot study.

## Conclusion

Data from the current pilot investigation indicate that short-term SymDyn indices may be used as reliable non-respiratory-associated parameters in laboratory settings to detect ANS activity modulations in elite modern male pentathlon athletes. These findings provide a potential solution for addressing the confounding influence of respiration frequency on HRV-derived inferences of cardiac autonomic function. For this reason, SymDyn may prove to be preferable for field-based monitoring where measurements are unsupervised.

## Data Availability Statement

The raw data supporting the conclusions of this article will be made available by the authors, without undue reservation.

## Ethics Statement

The studies involving human participants were reviewed and approved by University Ethical Committee (SKE 01-01/2017, 7 March 2017, Józef Piłsudski University of Physical Education in Warsaw, Warsaw, Poland). The patients/participants provided their written informed consent to participate in this study.

## Author Contributions

JG and BH: conceptualization and methodology. JG, MR, MM, BH, and RB: formal analysis and investigation. JG, MR, and MM: writing—original draft preparation. JG, MR, MM, AF, RB, and BW: writing—review and editing. All authors contributed to the article and approved the submitted version.

## Conflict of Interest

The authors declare that the research was conducted in the absence of any commercial or financial relationships that could be construed as a potential conflict of interest.

## Publisher’s Note

All claims expressed in this article are solely those of the authors and do not necessarily represent those of their affiliated organizations, or those of the publisher, the editors and the reviewers. Any product that may be evaluated in this article, or claim that may be made by its manufacturer, is not guaranteed or endorsed by the publisher.
